# Post-Treatment Trajectories of Retained Totally Implantable Venous Access Ports After Anticancer Therapy: A Conditional Landmark Cohort Study

**DOI:** 10.3390/curroncol33090525

**Published:** 2026-09-01

**Authors:** Zeyang Fan, Tiantian Li, Kai Yang

**Affiliations:** 1 Department of Interventional Vascular Surgery, Peking University First Hospital, Beijing 100034, China; fanzeyang@pku.edu.cn; 2 Department of Gastroenterology, Feicheng People’s Hospital, Feicheng 271600, China; 3 Department of Interventional Radiology, The Fourth Hospital of Hebei Medical University, Shijiazhuang 050011, China

**Keywords:** totally implantable venous access port (TIVAP), vascular access, TIVAP removal, TIVAP reactivation, TIVAP maintenance, competing risks, cancer survivorship, conditional landmark

## Abstract

**Background:** Retained totally implantable venous access ports (TIVAPs) pose a clinical management challenge after completion of intravenous anticancer therapy, particularly when future treatment needs remain uncertain. We aimed to characterize longitudinal post-treatment TIVAP trajectories after a day-90 conditional landmark, with elective removal prespecified as the primary first event. **Methods:** This retrospective cohort study included patients who were alive and event-free, retained the original TIVAP, had no documented TIVAP-related indication requiring removal, and had documented clinical and TIVAP status at day 90. Elective removal was the primary event; TIVAP reactivation, complication-related removal, and network-documented death were competing events. **Results:** Among 1406 patients, the 12-month cumulative incidence was 34.3% (95% confidence interval [CI], 31.4–37.1) for elective removal, 10.1% (95% CI, 8.2–11.9) for reactivation, 4.1% (95% CI, 3.0–5.3) for complication-related removal, and 1.0% (95% CI, 0.5–1.6) for network-documented death. During 1164.9 patient-years, 9978 maintenance-related patient-date encounters were recorded. In the assessed subcohort (*n* = 1319), 69.2% of assessments indicated willingness to choose a TIVAP again for similar future treatment, 64.4% indicated willingness to recommend TIVAP use, and 26.0% recorded a preference for earlier removal. **Conclusions:** Retained TIVAPs followed heterogeneous post-treatment courses, including elective removal, subsequent reuse, and continued retention requiring maintenance. These findings describe observed management patterns; they do not establish the comparative effectiveness of retention versus removal or identify an optimal removal time, and they support a prospective evaluation of structured reassessment pathways.

## Simple Summary

Totally implantable venous access ports (TIVAPs) are often retained after intravenous anticancer therapy because future treatment needs are uncertain. We studied 1406 patients who were alive and event-free, with the original TIVAP retained 90 days after their last qualifying intravenous anticancer treatment. By 12 months, 34.3% had elective removal, 10.1% reused the retained TIVAP for renewed intravenous anticancer treatment, and 4.1% underwent complication-related removal. Continued retention also required repeated maintenance. These findings describe heterogeneous post-treatment TIVAP trajectories but do not identify an optimal removal time; they support structured reassessment based on future treatment needs, TIVAP function, maintenance feasibility, future venous access, and patient preference.

## 1. Introduction

Totally implantable venous access ports (TIVAPs) provide reliable central venous access for repeated administration of systemic anticancer therapy, particularly when peripheral access is difficult or vesicant treatment is required [1,2]. After intravenous anticancer therapy is completed, however, the TIVAP may remain in place because the likelihood and timing of future treatment are uncertain. Clinicians must then balance the potential benefit of preserving reliable venous access against maintenance requirements, infection and thrombosis risk, TIVAP dysfunction, patient burden, and the possibility of difficult venous re-access after removal.

Previous studies have addressed patient experience with central venous access devices [3,4,5], post-treatment maintenance and flushing intervals [6,7,8,9,10,11], device utilization and complications during oncology care [12], and removal-related or longer-term TIVAP outcomes [13,14,15,16,17]. However, the post-treatment trajectory of a retained TIVAP—elective removal, renewed treatment use, complication-related removal, or continued retention—remains poorly characterized. Current oncology and vascular-access guidance supports individualized management based on anticipated future treatment, TIVAP function, complication risks, maintenance feasibility, future venous access options, and patient preference, but does not establish a universal removal time [6,18,19,20].

The primary objective was to characterize longitudinal post-treatment TIVAP trajectories after a prespecified day-90 conditional landmark among patients who remained alive and event-free with the original TIVAP retained. Elective TIVAP removal was prespecified as the primary first event within a competing-risk framework; TIVAP reactivation, complication-related removal, and network-documented death were competing events. Secondary objectives were to describe maintenance utilization, exploratory burden and management preferences, reactivation usability, and removal-related outcomes.

## 2. Materials and Methods

### 2.1. Study Design, Setting, and Data Sources

This retrospective longitudinal cohort study was conducted at Peking University First Hospital, a tertiary academic hospital in Beijing, China. The study was designed to characterize post-treatment management trajectories among patients with retained TIVAPs after completion of intravenous anticancer therapy. Patient-level longitudinal data were constructed by linking routinely collected institutional records, including intravenous anticancer treatment-administration records, TIVAP maintenance and thrombolysis records, outpatient and inpatient encounters, structured telephone or WeChat voice-call follow-up documentation, interventional and surgical removal records, complication records, and reimplantation records.

Follow-up information was obtained through routine clinical care pathways and was not initiated specifically for this study. Event classification was based on source-linked clinical records, with procedure and treatment-administration records prioritized over legacy summary fields when discrepancies were identified. Administrative-only activity did not extend clinical follow-up. Eligible final qualifying intravenous anticancer administrations occurred between 14 May 2020 and 2 October 2025, and corresponding day-90 landmarks occurred between 12 August 2020 and 31 December 2025. The latest active clinical contact included in the analytic dataset occurred on 22 July 2026. After completion of source reconciliation and temporal-consistency checks, the analytic database was locked on 23 July 2026; no records generated after the database lock were incorporated into the analyses. Reporting followed the Strengthening the Reporting of Observational Studies in Epidemiology (STROBE) and Reporting of studies Conducted using Observational Routinely collected health Data (RECORD) statements [21,22].

### 2.2. Cohort Entry and Conditional Landmark

Eligible source records represented adults with a solid tumor whose original TIVAP had been used for qualifying intravenous anticancer therapy and whose last qualifying administration date could be established. A qualifying administration was defined from linked institutional treatment-administration records as intravenous systemic anticancer therapy delivered through the original TIVAP. Maintenance-phase regimens were included when they consisted of intravenous systemic anticancer therapy administered through the original TIVAP. When multiple qualifying agents were administered on the same calendar date, the date was counted once, and the last such date was designated the index treatment date. Oral antineoplastic therapy and non-qualifying TIVAP uses—including premedication-only or supportive infusions, flushing, blood sampling, transfusion, contrast administration, hydration, and anti-infective treatment—did not define the index date or reactivation. Treatment administered outside the linked institutional network did not define the primary index date and was considered only during external-event sensitivity review.

**Figure 1:**
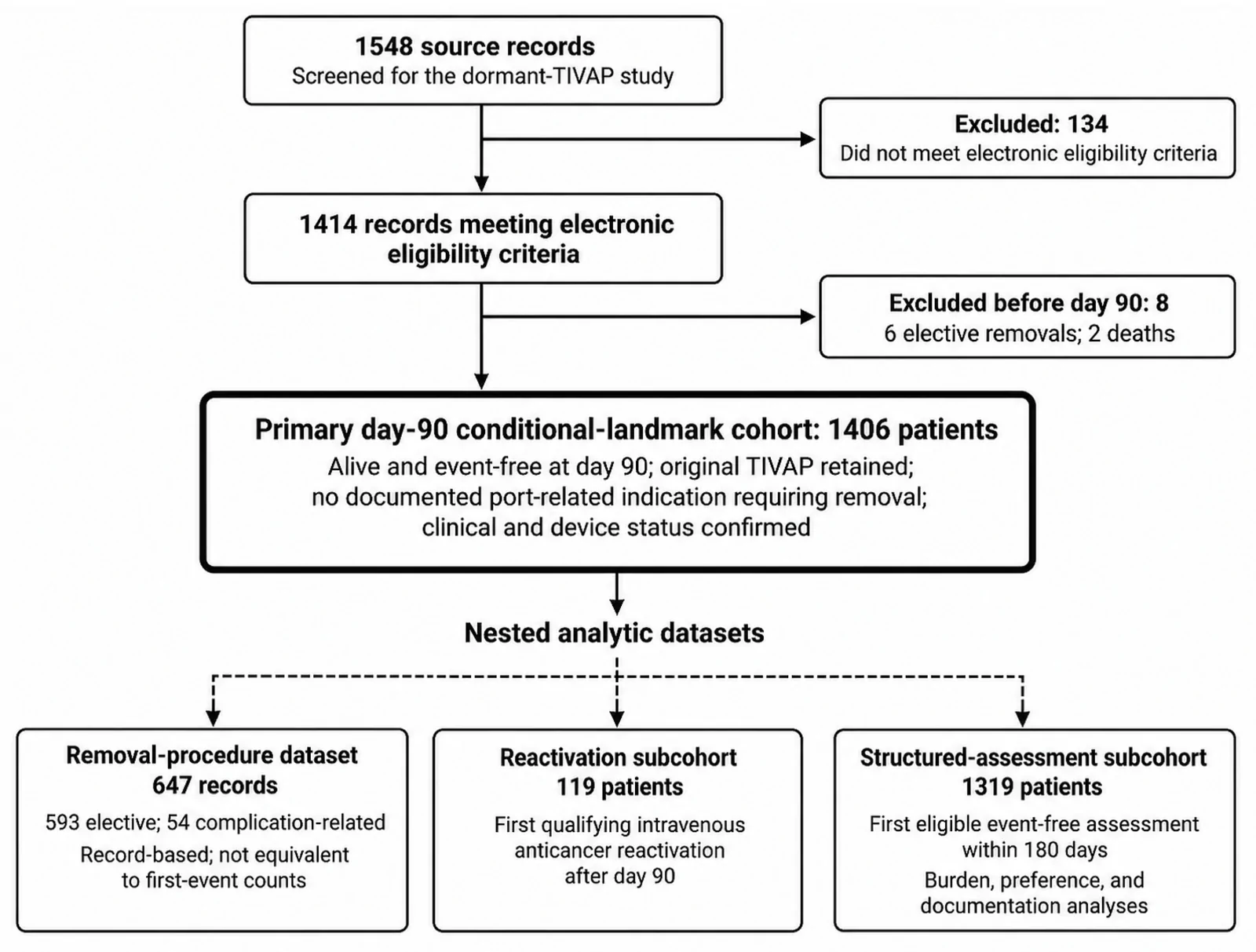
Cohort construction and nested analytic datasets. Of 1548 source records, 134 did not meet electronic eligibility criteria, and 1414 underwent pre-landmark assessment. Eight patients experienced a first event before day 90 (six elective removals and two deaths), leaving 1406 patients in the primary day-90 conditional-landmark cohort. The removal-procedure dataset was record-based and is not equivalent to patient-level first-event counts. The reactivation and structured-assessment datasets were nested subcohorts derived from the primary cohort. Dashed connectors denote nested analytic datasets rather than sequential exclusions. TIVAP, totally implantable venous access port.

The day-90 landmark was calculated from the last qualifying intravenous anticancer administration, not from the last maintenance or supportive-use record. To enter the primary risk set, a patient had to be alive, free of a first study event, retain the original TIVAP, have no documented TIVAP-related indication requiring removal, and have documented clinical and TIVAP status at day 90 (Figure 1; Supplementary Tables S1 and S2). A removal indication included infection, thrombosis, persistent occlusion or loss of function, mechanical failure, pocket or skin compromise, catheter fracture or damage, or another clinician-documented TIVAP-related condition requiring removal. The day-90 conditional landmark was selected a priori to identify patients entering a post-treatment management phase in which future intravenous therapy remained uncertain and decisions regarding continued TIVAP retention, reactivation, or elective removal became clinically relevant. At our institution, removal after treatment completion was not governed by a fixed interval; earlier removal could occur for a documented device-related indication, patient preference, or a clinical determination that future intravenous access was unlikely to be required. Among electronically eligible records, six elective removals and two deaths occurred before day 90. Day-60, day-120, and day-180 risk sets were reconstructed independently to assess sensitivity to landmark choice.

### 2.3. TIVAP Placement and Post-Treatment Maintenance Context

TIVAPs had been implanted before cohort entry for the administration of systemic anticancer therapy. Institutional image-guided chest-wall TIVAP implantation generally involved internal jugular venous access under real-time ultrasound guidance, fluoroscopic confirmation of the guidewire and catheter course, and final catheter-tip positioning near the cavoatrial junction. After completion of intravenous anticancer therapy, retained TIVAPs underwent routine maintenance during scheduled outpatient visits at our institution. In routine institutional practice, maintenance was generally scheduled at intervals ranging from 28 days to 3 months, according to the patient’s clinical circumstances and follow-up arrangements. Maintenance included inspection of the port site for local complications, assessment of blood return and infusion function, flushing, and a standardized port-lock procedure using heparinized saline at a concentration of 100 IU/mL [6]. Additional evaluation or thrombolytic management was performed when impaired blood return, occlusion, infection, thrombosis, or mechanical dysfunction was suspected.

### 2.4. Dormancy, Reactivation, and Vascular Access Status

For this study, the dormant interval referred specifically to the absence of qualifying intravenous anticancer administration through the original TIVAP; it did not imply that the TIVAP had no other clinical use. TIVAP reactivation was defined as the first subsequent qualifying intravenous anticancer administration through the retained original TIVAP. This endpoint documented renewed administration through the original TIVAP only; it did not establish durable resumption of treatment, normal TIVAP function in every respect, or continued use throughout later follow-up. Flushing, blood sampling, transfusion, contrast administration, hydration, anti-infective treatment, and other supportive intravenous uses did not constitute anticancer reactivation. Post-reactivation analyses described immediate treatment usability, thrombolysis, dysfunction, infection, thrombosis, occlusion, mechanical problems, and documented original TIVAP use during prespecified follow-up windows. Categories could overlap, and unconfirmed later status was retained as unknown rather than presumed absent.

### 2.5. Outcomes and Event Adjudication

The primary outcome was elective TIVAP removal, defined as removal performed in the absence of a documented TIVAP-related complication requiring removal. This category included planned or preference-driven removal after treatment completion when no complication was documented as the indication. Complication-related removal was defined as removal prompted by infection, thrombosis, persistent occlusion or loss of function, mechanical failure, pocket or skin compromise, catheter fracture or damage, or another documented TIVAP-related indication. Incidental or historical abnormalities that did not prompt removal did not change an otherwise elective classification. Other competing first events were TIVAP reactivation and network-documented death. Network-documented death was defined as all-cause death recorded in institutional information systems or confirmed through institutional follow-up. Other events identified from external records or patient/family reports were retained for sensitivity analyses and were not included in the primary endpoint. Patients without a documented first event were censored at the date of their last active clinical contact or the data-lock date, whichever occurred first; administrative-only activity did not extend follow-up.

Potentially conflicting dates were reconciled against procedure, treatment-administration, complication, and active-contact records. A final review identified ten death dates contained in legacy summary fields that were followed by later active clinical contact; five were reclassified to elective removal, three to complication-related removal, and two to censoring at the later confirmed contact. No same-day conflicting first events remained after source review; therefore, the prespecified hierarchy of complication-related removal, elective removal, reactivation, and network-documented death did not alter any final classification. The nested removal-procedure dataset was record-based and included six elective removals after prior reactivation; its record-level removal counts are therefore not directly comparable with patient-level first-event counts (Supplementary Tables S3 and S4).

### 2.6. Maintenance Utilization and Associated Recorded Charges

Maintenance-related records were restricted to the period from the day-90 landmark until the first documented event, censoring, or the data lock. A patient-date encounter was defined as one patient with at least one maintenance-related institutional record on a calendar date; multiple rows for the same patient and date were counted once in the total. Scheduled TIVAP maintenance, thrombolysis, unplanned TIVAP-related assessment, and evaluation for suspected dysfunction could coexist on one date. Category-specific counts were therefore non-mutually exclusive, whereas the total patient-date count was not duplicated. Recorded institutional charges associated with these encounters were summed in nominal Chinese yuan without inflation adjustment. These values represent hospital charge records, not the economic cost, payer expenditure, reimbursement, patient out-of-pocket payment, or societal burden.

### 2.7. Structured Assessment Subcohort

The assessed subcohort included the first eligible structured telephone, WeChat voice call, or clinic assessment within 180 days after the landmark and before any dated first event. These assessments were conducted as part of routine clinical follow-up and were not initiated specifically for this study. Ten locally developed, unvalidated subjective burden items addressed pain, foreign-body discomfort, body image, scar dissatisfaction, sleep, exercise, bathing, transportation, economic burden, and disease-reminder anxiety. The same items were asked directly of the respondent and rated as none, mild, moderate, or marked. Responses provided directly by patients were considered patient-reported, whereas responses provided by spouses or adult children were considered proxy-reported interpretations of the patient’s experience. Moderate or marked responses were used descriptively and did not represent validated clinical thresholds. The structured assessment also included direct questions regarding current TIVAP retention/removal preference, willingness to choose a TIVAP again for similar future treatment, and willingness to recommend TIVAP use.

“No explicit preference” denoted a completed assessment without a directional retention/removal preference, whereas “unrecorded” denoted absence of the field. Complete respondent-stratified burden, preference, clinician-recommendation, and agreement categories are reported in Supplementary Table S5.

### 2.8. Statistical Analysis

Aalen–Johansen estimators quantified the cumulative incidence of each first event in the presence of competing events. Pointwise 95% confidence intervals were obtained from 2000 patient-level bootstrap resamples, and the number at risk was defined as the number of patients who remained under observation without a documented first event at each displayed time point. Alternative landmark analyses independently reconstructed eligibility and reset follow-up at days 60, 120, and 180; these analyses describe different, progressively selected populations and do not estimate the effect of delaying reassessment. External-event scenarios assigned all 21 records without confirmed external-event status to elective removal, reactivation, or death at the censoring date as bounded sensitivity analyses. Agreement between classifiable patient and clinician positions was summarized using observed agreement, Cohen’s kappa, and Gwet’s AC1 with 2000 patient-level bootstrap resamples for confidence intervals. No adjusted association models were retained in the revised analysis. Analyses were performed in Python version 3.11.

## 3. Results

### 3.1. Cohort Construction and Baseline Characteristics

Among 1548 source records, 134 failed the electronic eligibility criteria related to age, tumor type, original-TIVAP use, date confirmation, record linkage, or duplicate episodes. The remaining 1414 records underwent pre-landmark assessment. Eight had a first event before day 90—six elective removals and two deaths—and all eight were excluded. All 1406 remaining patients had documented clinical and TIVAP status at the day-90 landmark and entered the primary conditional-landmark cohort (Figure 1; Supplementary Table S2). The median follow-up after the landmark was 242 days (IQR, 139–417). The cohort included 901 women (64.1%), and 543 patients (38.6%) lived outside Beijing. Thoracic, gastrointestinal, and breast cancers accounted for 30.9%, 24.4%, and 21.1%, respectively (Table 1).

### 3.2. Cumulative Incidence of First Post-Treatment TIVAP Events

**Figure 2:**
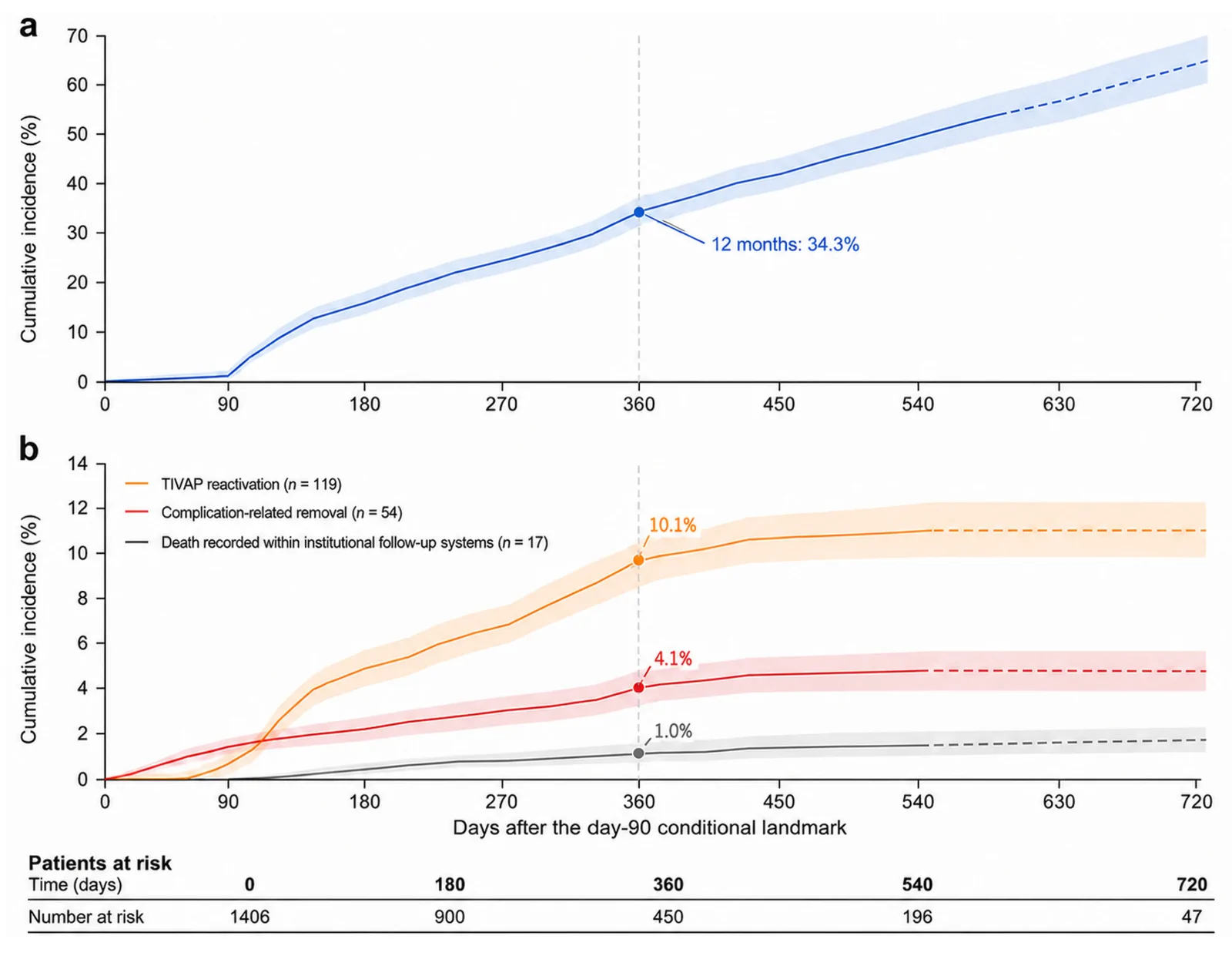
Cumulative incidence of post-treatment outcomes among patients with retained totally implantable venous access ports (TIVAPs) after intravenous anticancer therapy. (**a**) Elective TIVAP removal after the day-90 conditional landmark. (**b**) Competing first events, including TIVAP reactivation, complication-related removal, and death recorded within institutional follow-up systems. Curves were estimated using the Aalen–Johansen method; shaded areas represent 95% confidence intervals, dashed segments indicate follow-up beyond 18 months, and the risk table presents patients remaining event-free and under observation.

During all available follow-up, 587 patients underwent elective removal, 119 had documented TIVAP reactivation, 54 underwent complication-related removal, 17 had network-documented death, and 629 were censored without a documented first event. At 12 months, cumulative incidence was 34.3% (95% CI, 31.4–37.1) for elective removal, 10.1% (8.2–11.9) for reactivation, 4.1% (3.0–5.3) for complication-related removal, and 1.0% (0.5–1.6) for network-documented death (Figure 2; Supplementary Table S3). The 18-month elective-removal estimate was 51.2% (48.0–54.5). Only 47 patients remained at risk at 720 days; estimates beyond 18 months were therefore considered imprecise and were not emphasized.

### 3.3. Maintenance Utilization

From the day-90 landmark until the first documented event, censoring, or the data lock, 9978 patient-date maintenance encounters were recorded over 1164.9 patient-years of observation, equivalent to 8.57 encounters per patient-year. Patients had a median of six encounters (IQR, 3–10); nine (0.6%) had no documented maintenance encounter. The total included scheduled maintenance and problem-driven contacts. There were 325 thrombolysis encounters, 433 unplanned TIVAP-related assessments, and 492 evaluations for suspected TIVAP dysfunction. These categories could overlap on the same date and should not be summed to reproduce the total encounter count. Recorded institutional charges associated with included encounters were summarized descriptively. These records represent institutional billing data only and should not be interpreted as economic costs, payer expenditure, patient payments, or societal burden.

### 3.4. Structured Burden Assessment and Management Preferences After Treatment Completion

**Figure 3:**
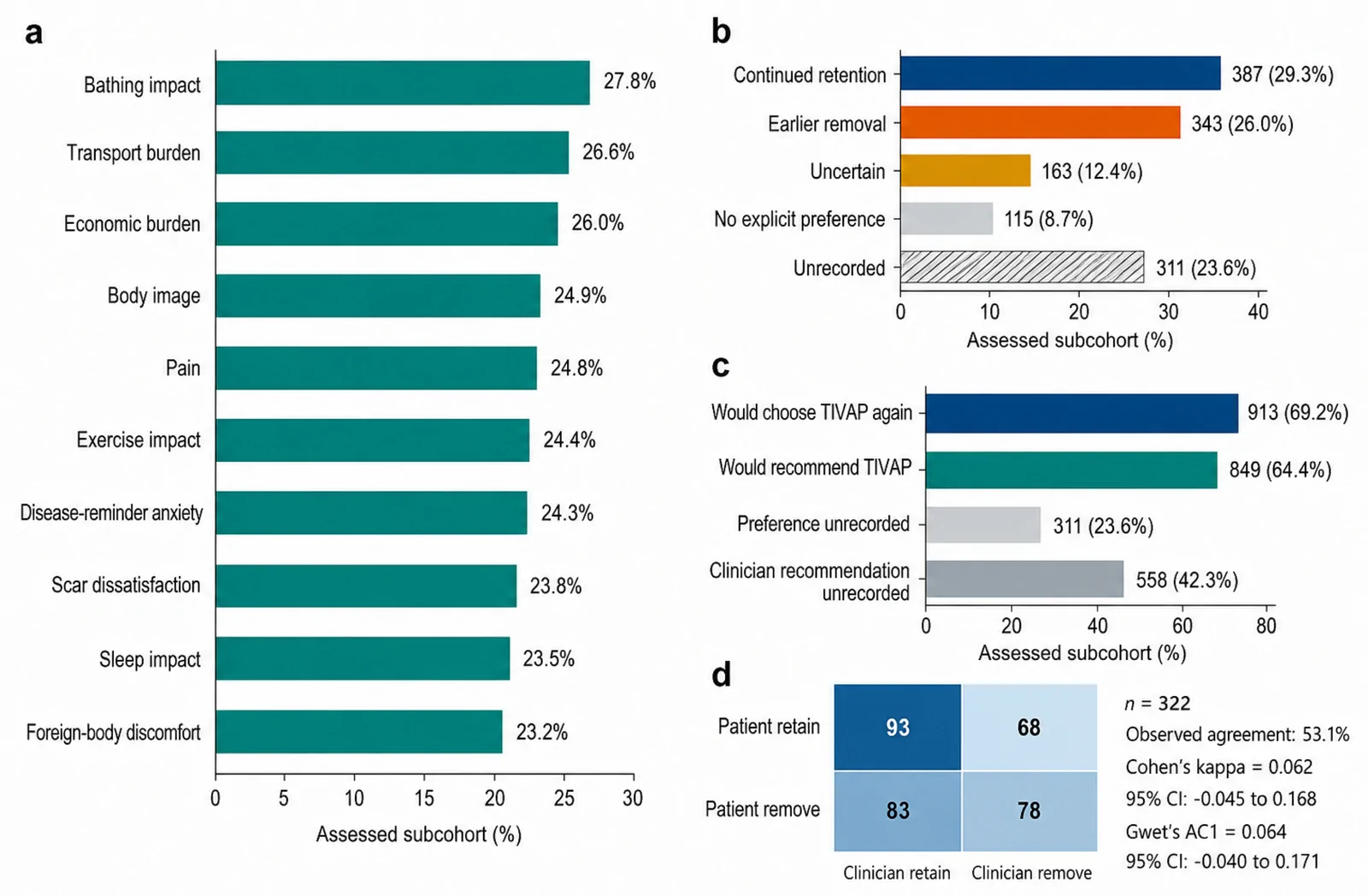
Exploratory burden assessment, management preferences, and patient–clinician concordance after treatment completion. (**a**) Exploratory TIVAP-related burden domains among the event-free assessed subcohort (*n* = 1319). Bars show pooled proportions of moderate or marked responses across direct patient and proxy assessments using locally developed, unvalidated subjective burden items; respondent-stratified results are provided in Supplementary Figure S1. (**b**) Current documented TIVAP-management preferences recorded near the assessment date. Categories were mutually exclusive and included continued retention, earlier removal, uncertain preference, no explicit preference, and unrecorded preference. (**c**) Treatment-phase acceptability and documentation completeness. Responses indicating willingness to choose a TIVAP again for similar future treatment and willingness to recommend TIVAP use reflect retrospective treatment-phase acceptability rather than current management preference. (**d**) Patient–clinician management concordance among paired directional records (*n* = 322). Values in the matrix represent the number of assessments classified according to patient preference and clinician recommendation regarding TIVAP retention or removal.

Structured burden and preference assessments were available for 1319 event-free patients at a median of 111 days (IQR, 92–134) after the day-90 landmark. Patients responded directly in 653 assessments (49.5%), while spouses or adult children responded as proxies in 666 (50.5%). Moderate or marked responses across the ten unvalidated subjective burden items ranged from 23.2% for foreign-body discomfort to 27.8% for bathing impact (Figure 3). Respondent-stratified patterns were generally similar; however, proxy responses represent the proxy respondents’ interpretation of the patients’ subjective experience and should not be interpreted as direct patient-reported responses (Supplementary Figure S1 and Table S5).

Continued retention was recorded in 387 assessments (29.3%), earlier removal in 343 (26.0%), uncertainty in 163 (12.4%), and no explicit preference in 115 (8.7%); preference was unrecorded in 311 (23.6%). In 913 assessments (69.2%), responses indicated willingness to choose a TIVAP again for similar future treatment, and 849 (64.4%) indicated willingness to recommend TIVAP use. These responses reflect retrospective treatment-phase acceptability rather than current preference regarding management of the retained TIVAP.

Clinician recommendation was unrecorded in 558 assessments (42.3%). Among the 322 records with classifiable patient and clinician positions, observed agreement was 53.1%, Cohen’s kappa was 0.062 (95% bootstrap CI, −0.045 to 0.168), and Gwet’s AC1 was 0.064 (95% bootstrap CI, −0.040 to 0.171) (Supplementary Table S5). The low chance-adjusted agreement should be interpreted in the context of incomplete retrospective documentation rather than as evidence regarding the quality of shared decision-making.

### 3.5. TIVAP Reactivation Patterns and Subsequent Removal-Related Outcomes

Among 119 first reactivations, the retained original TIVAP was documented as functional for treatment administration in 106 (89.1%), and thrombolysis was documented in 21 (17.6%); these categories were not mutually exclusive. During the subsequent 180-day observation period, documented device-related findings included dysfunction in 13 patients, infection in 5, thrombosis in 8, occlusion in 21, and mechanical problems in 3; individual patients could contribute more than one category. Subsequent institutional records documented original-TIVAP use during the 90-day follow-up window in 82 patients and during the 180-day window in 45; later use status was not confirmable in 37 and 74 patients, respectively. Reactivation was defined specifically as renewed intravenous anticancer treatment delivered through the retained original TIVAP. Oral antineoplastic therapy and treatments delivered outside the linked institutional network were not captured; therefore, the observed 12-month reactivation incidence should not be interpreted as a measure of overall anticancer-treatment continuation or disease control (Supplementary Table S6; Supplementary Figure S2).

The record-based removal dataset contained 647 removal records, including 593 elective and 54 complication-related removals. Because six elective removals occurred after prior reactivation, these record-level removal counts represent procedures rather than mutually exclusive patient-level first events. Difficult extraction was documented in 62 records (9.6%), catheter integrity abnormalities in 30 (4.6%), and procedural or same-day removal-related events in 16 (2.5%); component definitions are provided in Supplementary Table S4. Later reimplantation was documented in 15 records (2.3%). Complete 30-day post-removal ascertainment was available for 267 records; no new complication was documented within this subset. Because 380 records lacked complete ascertainment, these findings were not used to estimate an overall 30-day complication incidence. Post-removal oncologic treatment trajectories, particularly transitions to oral therapy or treatment delivered outside the linked network, could not be fully reconstructed (Supplementary Table S4; Supplementary Figure S3).

### 3.6. Sensitivity Analyses

Independently reconstructed alternative conditional-landmark risk sets included 1414, 1406, 1399, and 1338 patients at days 60, 90, 120, and 180, respectively. The 12-month cumulative incidence of elective removal ranged from 31.9% in the day-60 risk set to 42.6% in the day-180 risk set, whereas reactivation ranged from 8.9% to 10.9% (Supplementary Figure S4 and Table S7). Because eligibility was independently re-evaluated at each landmark, these estimates represent different conditional cohorts rather than a temporal comparison of reassessment strategies.

An external-event review was attempted for 615 of 629 censored records. Eight external events were confirmed (four reactivations, two elective removals, and two deaths), seven records remained unverifiable, and fourteen were not reviewed. Assigning the 21 records without confirmed external-event status to elective removal, reactivation, or death at the censoring date yielded bounded 12-month scenario estimates of up to 34.8%, 10.7%, and 1.6%, respectively. The death-assignment scenario should not be interpreted as network-documented mortality. Residence-stratified cumulative-incidence estimates are presented descriptively and were not intended as adjusted comparisons (Supplementary Table S7 and Figure S5).

## 4. Discussion

### 4.1. Principal Findings

In this selected day-90 cohort, elective TIVAP removal occurred in 34.3% by 12 months and 51.2% by 18 months, while reactivation through the retained original TIVAP occurred in 10.1% by 12 months and complication-related removal in 4.1%. Retention was associated with repeated vascular-access maintenance encounters, and a subset required thrombolysis or evaluation for suspected dysfunction. These findings describe heterogeneous post-treatment TIVAP courses in patients who had already remained alive and event-free with the original TIVAP retained through day 90. The study does not compare retention with removal; the findings support structured reassessment rather than a uniform removal-timing rule.

The exploratory assessments provided contextual information on burden and preferences. Responses indicated willingness to choose a TIVAP again for similar future treatment in 69.2% of assessments and willingness to recommend TIVAP use in 64.4%, whereas 26.0% of assessments recorded a preference for earlier removal. For proxy-completed assessments, these responses represent the proxy respondent’s interpretation rather than a direct patient report. These findings distinguish treatment-phase acceptability from current preference regarding retention of the existing TIVAP [3,4,5].

### 4.2. Clinical Interpretation and Treatment Trajectories

Previous work has described central venous access utilization, complications, and additional hospital visits during active systemic therapy [12]. The present study extends observation into the post-treatment period, when management shifts toward balancing TIVAP patency, infection and thrombosis risk, maintenance feasibility, readiness for renewed intravenous therapy, elective removal, and the technical feasibility of future venous re-access. This phase is therefore better viewed as longitudinal vascular-access stewardship than as a fixed extension of the implantation episode.

International evidence provides broader context for this post-treatment phase. ESMO and ASCO guidance emphasize ongoing review of the indication for central venous access, device function, complication surveillance, and removal when the device is no longer clinically required [19,20]. Prospective and longitudinal studies from Italy, Germany, and Turkey have documented late events during flushing-only periods, high but incomplete patient satisfaction, and prolonged device availability during long-term follow-up [15,16,17]. Together, these geographically diverse data support individualized longitudinal assessment, but they likewise do not establish a universal timing rule for elective TIVAP removal.

The low observed reactivation rate requires cautious interpretation. It does not demonstrate high anticancer efficacy because reactivation was defined only as renewed intravenous anticancer treatment through the retained original TIVAP and did not capture oral therapy or all out-of-network treatment. When reactivation did occur, 106 of 119 TIVAPs were immediately usable, but 21 required thrombolysis and later TIVAP-related problems were documented. Removal was also not uniformly trivial: difficult extraction occurred in 62 of 647 removal records, with additional catheter-integrity abnormalities and same-day procedural events. These findings support reassessing both current TIVAP function and future access options rather than assuming that either retention or removal is uniformly preferable.

### 4.3. Maintenance Burden, Preferences, and Decision Documentation

Retention generated 9978 documented maintenance-related patient-date encounters over 1164.9 patient-years. Recorded hospital charges capture only institutional billing and not transportation, time away from work, caregiver time, anxiety, or out-of-network care. Rather than supporting a formal economic comparison, these data indicate that continued retention was associated with recurrent care contacts during a period in which future intravenous treatment was uncertain.

The exploratory burden findings should be interpreted cautiously because the items were locally developed and unvalidated, 50.5% of assessments were proxy-completed, and patients with earlier events were excluded. Patient preference and clinician recommendation were also frequently unrecorded, limiting retrospective reconstruction of concordance. Prospective studies should use validated vascular-access measures and prospectively document anticipated future intravenous treatment, TIVAP function, maintenance feasibility, patient preference, clinician recommendation, review date, and contingency planning.

### 4.4. Strengths, Limitations, and Implications

Strengths include a large longitudinal cohort, an explicit conditional landmark, competing-risk estimation, independently reconstructed alternative landmarks, source-linked maintenance and procedural records, analysis-specific denominators, and temporal-consistency review. The principal contribution is a longitudinal description of retained-TIVAP outcomes, maintenance utilization, exploratory burden assessments from patient and proxy respondents, and documentation gaps after completion of intravenous anticancer therapy; this study does not provide a decision rule. Important limitations include the single-center setting; selection created by entry into a day-90 conditional risk set; incomplete ascertainment of death, TIVAP events, and treatment delivered outside the institutional network; unvalidated burden items with 50.5% proxy responses; non-uniform encoding of individual-level adherence to the intended maintenance schedule and of exact flushing and locking volumes in the retrospective records; recorded charges that do not represent the full economic costs; and incomplete 30-day follow-up after removal. This study also could not comprehensively reconstruct treatment after elective removal, including transitions to oral anticancer therapy or external intravenous treatment. Consequently, the 10.1% reactivation estimate should not be used to infer disease control or to justify routine earlier removal. These limitations constrain generalizability and causal interpretation and support prospective multicenter evaluation of structured reassessment integrating anticipated timing of future intravenous therapy, blood return and infusion function, complication concerns, maintenance feasibility, anticipated difficulty with future venous access, patient preference, and a contingency plan.

## 5. Conclusions

In this selected cohort, retained TIVAPs demonstrated heterogeneous post-treatment trajectories, including elective removal, subsequent reuse, and continued retention requiring maintenance. These findings describe observed management patterns after intravenous anticancer therapy; they do not compare retention and removal strategies or identify an optimal removal time. Prospective multicenter studies should evaluate structured reassessment pathways integrating future intravenous treatment needs, TIVAP function, complication risk, maintenance feasibility, and patient preference.

## Figures and Tables

**Table 1:** Baseline characteristics of the day-90 conditional-landmark cohort.

Characteristic	Value
Age, years	63.0 (50.0–75.0)
Female sex	901 (64.1%)
Body mass index, kg/m^2^	23.6 (20.5–26.4)
Residence outside Beijing	543 (38.6%)
Charlson Comorbidity Index	2.0 (1.0–4.0)
Diabetes mellitus	245 (17.4%)
Previous venous thromboembolism	81 (5.8%)
Metastatic disease at landmark	634 (45.1%)
Anticoagulation at landmark	49 (3.5%)
TIVAP age at the day-90 landmark, months	10.0 (8.0–11.9)
TIVAP-related complication during active treatment	69 (4.9%)
Cancer group: breast	297 (21.1%)
Cancer group: thoracic	435 (30.9%)
Cancer group: gastrointestinal	343 (24.4%)
Cancer group: hepatobiliary/pancreatic	103 (7.3%)
Cancer group: gynecologic	95 (6.8%)
Cancer group: urologic	52 (3.7%)
Cancer group: head and neck	32 (2.3%)
Cancer group: other solid tumors	49 (3.5%)
Treatment intent: adjuvant	482 (34.3%)
Treatment intent: neoadjuvant	247 (17.6%)
Treatment intent: curative	157 (11.2%)
Treatment intent: palliative	429 (30.5%)
Treatment intent: maintenance	91 (6.5%)

Data are presented as median (IQR) or *n* (%). No values were missing for the variables shown. BMI, body mass index; IQR, interquartile range; TIVAP, totally implantable venous access port.

## Data Availability

De-identified data supporting the findings of this study are available from the corresponding author upon reasonable request, subject to institutional approval and applicable data protection regulations.

## Supplementary Materials

The following supporting information can be downloaded at: https://www.mdpi.com/article/10.3390/curroncol33090525/s1, Supplementary File S1: Supplementary Methods (including Table S1: Operational definitions of study events and outcomes; Table S2: Cohort construction and analytic populations; Table S3: Longitudinal first events after the day-90 landmark; Table S4: Removal-procedure characteristics and post-removal outcomes; Table S5: Patient- and proxy-reported burden and management preferences; Table S6: Clinical characteristics and outcomes after TIVAP reactivation; Table S7: Sensitivity analyses summary; Supplementary Figure S1: Exploratory TIVAP-related burden domains stratified by respondent type; Supplementary Figure S2: Characteristics of first TIVAP reactivation and subsequent documented use of the retained original TIVAP; Supplementary Figure S3: Record-based removal outcomes and completeness of post-removal outcome ascertainment; Supplementary Figure S4: Alternative conditional-landmark analyses of TIVAP management trajectories; Supplementary Figure S5: Sensitivity analyses for unresolved external-event scenarios and residence-stratified cumulative incidence estimates); Supplementary File S2: De-identified aggregate outputs, operational definitions, selected figure-generation code, and locked-output verification code.

## Abbreviations

**Abbreviation**	**Definition**
AC1	Gwet’s agreement coefficient 1
BMI	Body mass index
CI	Confidence interval
IQR	Interquartile range
RECORD	Reporting of studies Conducted using Observational Routinely collected health Data
STROBE	Strengthening the Reporting of Observational Studies in Epidemiology
TIVAP	Totally implantable venous access port
