# Supplementary material for: Post-Treatment Trajectories of Retained Totally Implantable Venous Access Ports After Anticancer Therapy: A Conditional Landmark Cohort Study
Source: Curr Oncol. 2026 Sep 1;33(9):525. doi: 10.3390/curroncol33090525 (PMC13605111; doi:10.3390/curroncol33090525)
Supplement: Supplementary file 1 [file curroncol-33-00525-s001.zip › curroncol-4488959-Supplementary File S1.pdf]

Supplementary Materials

Supplementary Methods

The supplementary analyses used the same locked day-90 conditional-landmark cohort and first-event definitions as the main manuscript. Alternative day-60, day-120, and day-180 cohorts were independently reconstructed. Cumulative incidence was estimated with the Aalen–Johansen method in the presence of competing events, with pointwise 95% confidence intervals from 2000 patient-level bootstrap resamples. Structured burden analyses used the first eligible assessment within 180 days after the landmark and before any dated first event. Reactivation and removal-procedure summaries used their analysis-specific denominators; overlapping categories are identified. No adjusted association models are included in the revised supplementary materials.

Supplementary Table S1. Operational definitions of study events and outcomes

Variable	Operational definition
Day-90 risk set	Alive and free of a first study event; original TIVAP retained; no documented TIVAP-related indication requiring removal; clinical and TIVAP status documented at day 90
Elective removal	Removal in the absence of a documented TIVAP-related complication requiring removal
TIVAP reactivation	First subsequent qualifying intravenous anticancer administration through the retained original TIVAP; does not imply durable treatment resumption or normal long-term TIVAP function
Complication-related removal	Removal prompted by infection, thrombosis, persistent occlusion or loss of function, mechanical failure, pocket or skin compromise, catheter fracture or damage, or another documented TIVAP-related indication
Network-documented death	All-cause death recorded in institutional information systems or confirmed through the institutional follow-up network
Censoring	Last active clinical contact or administrative data lock, whichever occurred first
Maintenance encounter	One patient with at least one maintenance-related institutional record on a calendar date between the landmark and first event or censoring
Difficult extraction	Removal record explicitly classified as difficult extraction in the source procedure documentation; technical difficulty was not graded retrospectively
Catheter integrity abnormality	Documented catheter damage, fracture, or another integrity abnormality in the removal record
Removal-related event	Documented procedural or same-day removal-related event

Supplementary Table S2. Cohort construction and analytic populations

Stage or analytic population	n	Definition or reason
Source records	1548	Potential qualifying dormant-TIVAP episodes
Electronic exclusions	134	Failed age, tumor, original-TIVAP use, date confirmation, linkage, or duplicate criteria
Electronically eligible	1414	Entered pre-landmark assessment
Pre-day-90 first events	8	Six elective removals and two deaths; excluded
Day-90 cohort	1406	Primary conditional-landmark risk set
Removal-procedure dataset	647 records	593 elective and 54 complication-related removals; includes six elective removals after reactivation
Reactivation subcohort	119 patients	Patients whose first event was reactivation through the retained original TIVAP
Structured-assessment subcohort	1319 assessments	First eligible event-free assessment within 180 days after the landmark

Supplementary Table S3. Longitudinal first events after the day-90 landmark

First event	Total n	6-month CIF (95% CI)	12-month CIF (95% CI)	18-month CIF (95% CI)
Elective removal	587	16.2% (14.1–18.2)	34.3% (31.4–37.1)	51.2% (48.0–54.5)
TIVAP reactivation	119	4.2% (3.1–5.3)	10.1% (8.2–11.9)	10.9% (9.0–12.9)
Complication-related removal	54	2.3% (1.5–3.1)	4.1% (3.0–5.3)	4.8% (3.5–6.1)
Network-documented death	17	0.3% (0.1–0.6)	1.0% (0.5–1.6)	1.4% (0.7–2.2)

Patients censored without a documented first event: 629. Numbers at risk at exact days 0, 180, 360, 540, and 720 were 1406, 900, 450, 196, and 47, respectively. CIF, cumulative incidence function; CI, confidence interval.

Supplementary Table S4. Removal-procedure characteristics and post-removal outcomes

Measure	n (%)	Interpretive note
Removal records	647	Record-based denominator
Elective removal records	593 (91.7%)	Includes six elective removals after prior reactivation
Complication-related removal records	54 (8.3%)	Procedure classification
Difficult extraction documented	62 (9.6%)	Source-record classification; technical difficulty not graded
Catheter integrity abnormality	30 (4.6%)	Damage, fracture, or another recorded integrity abnormality
Procedural or same-day removal-related event	16 (2.5%)	Removal-related event category
Later reimplantation	15 (2.3%)	Documented in institutional records
Complete 30-day post-removal ascertainment	267 (41.3%)	No new post-day-0 complication documented in this subset
Incomplete 30-day ascertainment	380 (58.7%)	Precludes estimation of an overall 30-day complication incidence

Supplementary Table S5. Patient- and proxy-reported burden and management preferences

Panel A. Moderate or marked responses on locally developed, unvalidated burden items

Burden item	Overall (n = 1319)	Patient respondent (n = 653)	Proxy respondent (n = 666)
Pain	327/1319 (24.8%)	163/653 (25.0%)	164/666 (24.6%)
Foreign-body discomfort	306/1319 (23.2%)	157/653 (24.0%)	149/666 (22.4%)
Body image	328/1319 (24.9%)	163/653 (25.0%)	165/666 (24.8%)
Scar dissatisfaction	314/1319 (23.8%)	173/653 (26.5%)	141/666 (21.2%)
Sleep impact	310/1319 (23.5%)	148/653 (22.7%)	162/666 (24.3%)
Exercise impact	322/1319 (24.4%)	159/653 (24.3%)	163/666 (24.5%)

Burden item	Overall (n = 1319)	Patient respondent (n = 653)	Proxy respondent (n = 666)
Bathing impact	367/1319 (27.8%)	178/653 (27.3%)	189/666 (28.4%)
Transport burden	351/1319 (26.6%)	164/653 (25.1%)	187/666 (28.1%)
Economic burden	343/1319 (26.0%)	156/653 (23.9%)	187/666 (28.1%)
Disease-reminder anxiety	321/1319 (24.3%)	165/653 (25.3%)	156/666 (23.4%)

Panel B. Preferences, clinician recommendations, and treatment-phase acceptability

Field	Category	n (%) of 1319
Patient preference	Continued retention	387 (29.3%)
Patient preference	Earlier removal	343 (26.0%)
Patient preference	Uncertain	163 (12.4%)
Patient preference	No explicit preference	115 (8.7%)
Patient preference	Unrecorded	311 (23.6%)
Clinician recommendation	Recommend retention	310 (23.5%)
Clinician recommendation	Recommend removal	221 (16.8%)
Clinician recommendation	Either/shared discussion	88 (6.7%)
Clinician recommendation	Defer decision	142 (10.8%)
Clinician recommendation	Unrecorded	558 (42.3%)
Treatment-phase acceptability	Would choose a TIVAP again	913 (69.2%)
Treatment-phase acceptability	Would recommend TIVAP use	849 (64.4%)

Panel C. Patient–clinician directional agreement among 322 classifiable pairs

Patient preference	Clinician: remove	Clinician: retain
Patient: remove	78	83
Patient: retain	68	93

Observed agreement, 53.1%; Cohen's kappa, 0.062 (95% bootstrap CI, −0.045 to 0.168); Gwet's AC1, 0.064 (95% bootstrap CI, −0.040 to 0.171). Proxy responses represent proxy respondents' interpretations of patients' subjective experiences and should not be interpreted as direct patient-reported responses.

Supplementary Table S6. Clinical characteristics and outcomes after TIVAP reactivation

Measure	n (%)	Interpretive note
First reactivations	119	Analysis denominator
Immediately usable for treatment administration	106 (89.1%)	May overlap with thrombolysis
Thrombolysis documented	21 (17.6%)	May overlap with immediate usability
Functional dysfunction within 180 days	13 (10.9%)	Device-related findings are non-mutually exclusive
Infection within 180 days	5 (4.2%)	Non-mutually exclusive
Thrombosis within 180 days	8 (6.7%)	Non-mutually exclusive
Occlusion within 180 days	21 (17.6%)	Non-mutually exclusive
Mechanical problem within 180 days	3 (2.5%)	Non-mutually exclusive
Original-TIVAP use documented by day 30	119 (100.0%)	Reactivation administration
Original-TIVAP use documented by day 90	82 (68.9%)	Status not confirmed in 37
Original-TIVAP use documented by day 180	45 (37.8%)	Status not confirmed in 74

Supplementary Table S7. Sensitivity analyses summary

Panel A. Independently reconstructed conditional landmarks

Landmark	Risk set n	Elective removal	Reactivation	Complication removal	Network death
Day 60	1414	31.9% (29.3–34.5)	8.9% (7.3–10.6)	3.7% (2.7–4.8)	1.0% (0.5–1.7)
Day 90	1406	34.3% (31.4–37.1)	10.1% (8.2–11.9)	4.1% (3.0–5.3)	1.0% (0.5–1.6)
Day 120	1399	36.8% (33.7–39.6)	10.7% (8.9–12.6)	4.6% (3.3–5.9)	1.0% (0.5–1.6)
Day 180	1338	42.6% (39.4–45.6)	10.9% (9.1–12.8)	3.7% (2.5–4.9)	1.1% (0.5–1.8)

Values are 12-month cumulative incidence (95% CI). Risk sets differ by landmark and are not causal comparisons of reassessment timing.

Panel B. External-event review and bounded scenarios

Scenario or review status	Value	Interpretation
Censored records	629	Primary cohort
External-event review attempted	615	Among censored records
Confirmed external events	8	Four reactivations, two elective removals, and two deaths
Unverifiable	7	External status not confirmed
Not reviewed	14	External review unavailable
All 21 assigned to elective removal	34.8%	Upper bounded 12-month elective-removal estimate
All 21 assigned to reactivation	10.7%	Upper bounded 12-month reactivation estimate
All 21 assigned to death	1.6%	Bounded scenario; not network-documented mortality

Supplementary Figure Legends

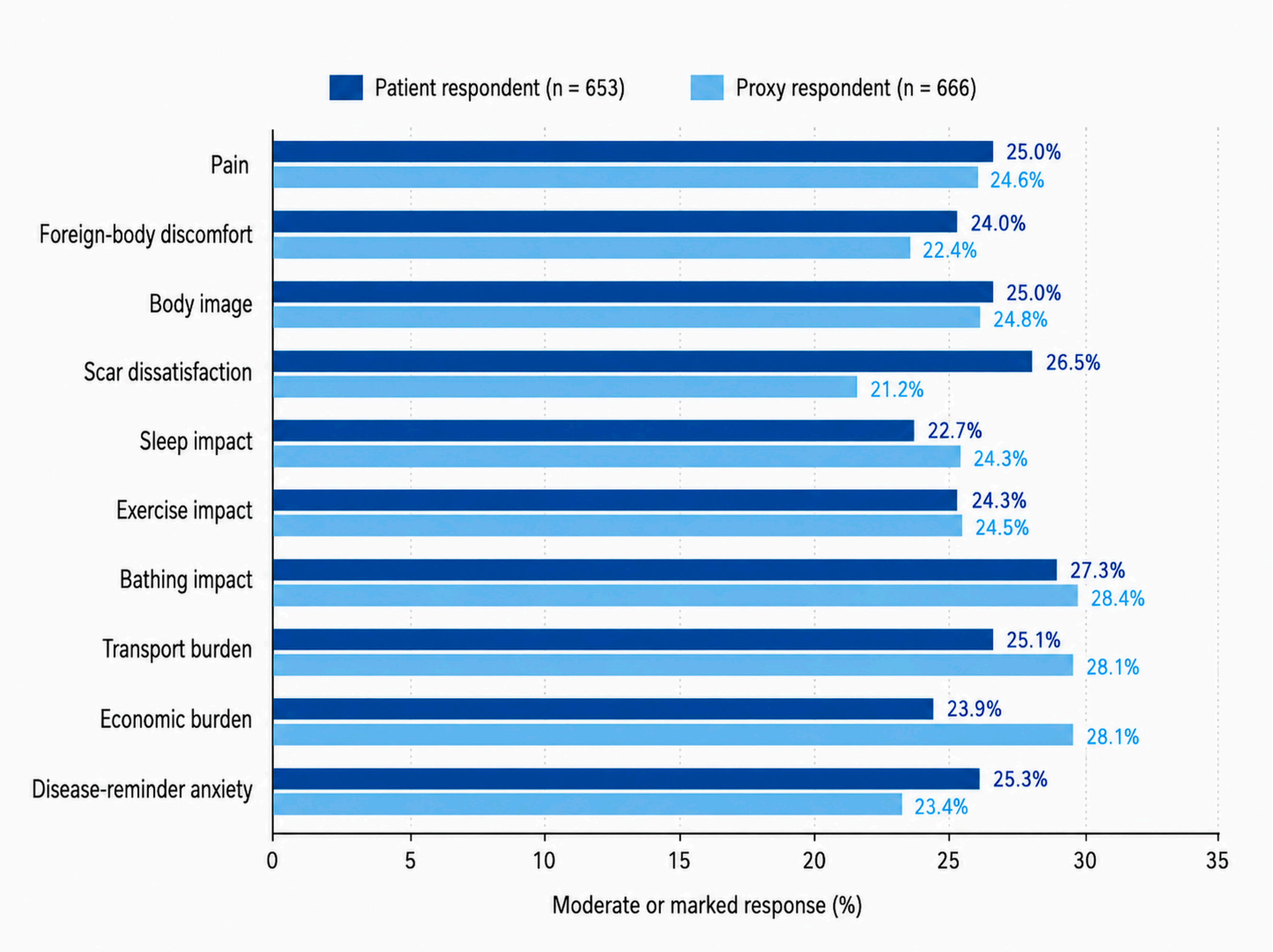

Supplementary Figure S1. Exploratory TIVAP-related burden domains stratified by respondent type. Moderate or marked responses across ten locally structured, unvalidated TIVAP-related burden domains are presented separately for direct patient respondents and proxy respondents. Assessed domains included pain, foreign-body discomfort, body image concerns, scar dissatisfaction, sleep impact, exercise impact, bathing impact, transport burden, economic burden, and disease-reminder anxiety. Proxy responses were analyzed descriptively; they represent proxy respondents' interpretations of patients' subjective experiences and should not be interpreted as direct patient-reported responses.

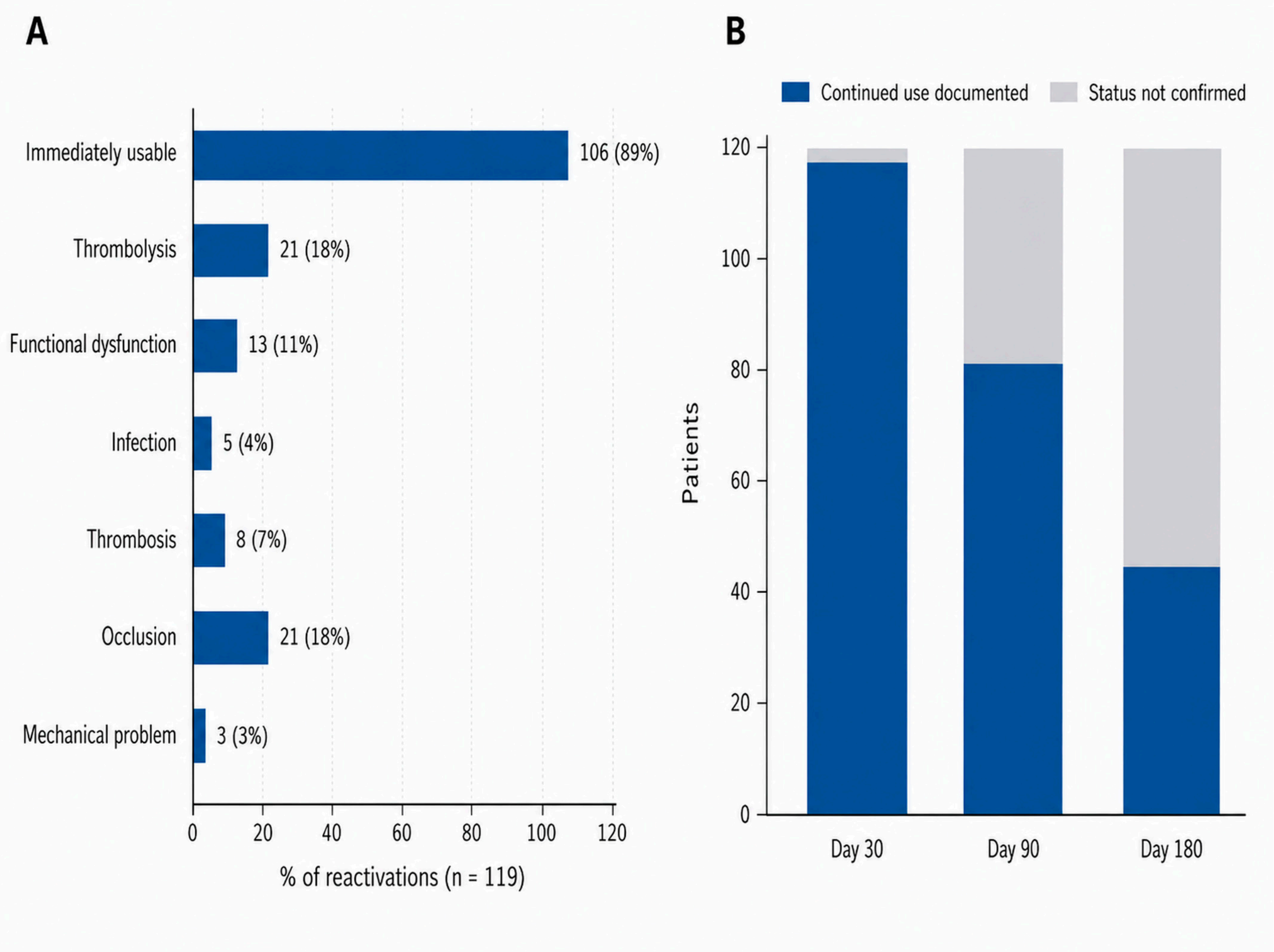

Supplementary Figure S2. Characteristics of first TIVAP reactivation and subsequent documented use of the retained original TIVAP. (A) Documented characteristics associated with first TIVAP reactivation, including immediate usability for treatment administration and device-related management interventions. Categories were not mutually exclusive; individual patients could contribute to more than one category. (B) Subsequent documented use status of the retained original TIVAP after reactivation during predefined follow-up windows. Continued use was classified according to available institutional treatment-administration records. Patients without confirmable subsequent use were classified as unavailable status rather than discontinued use.

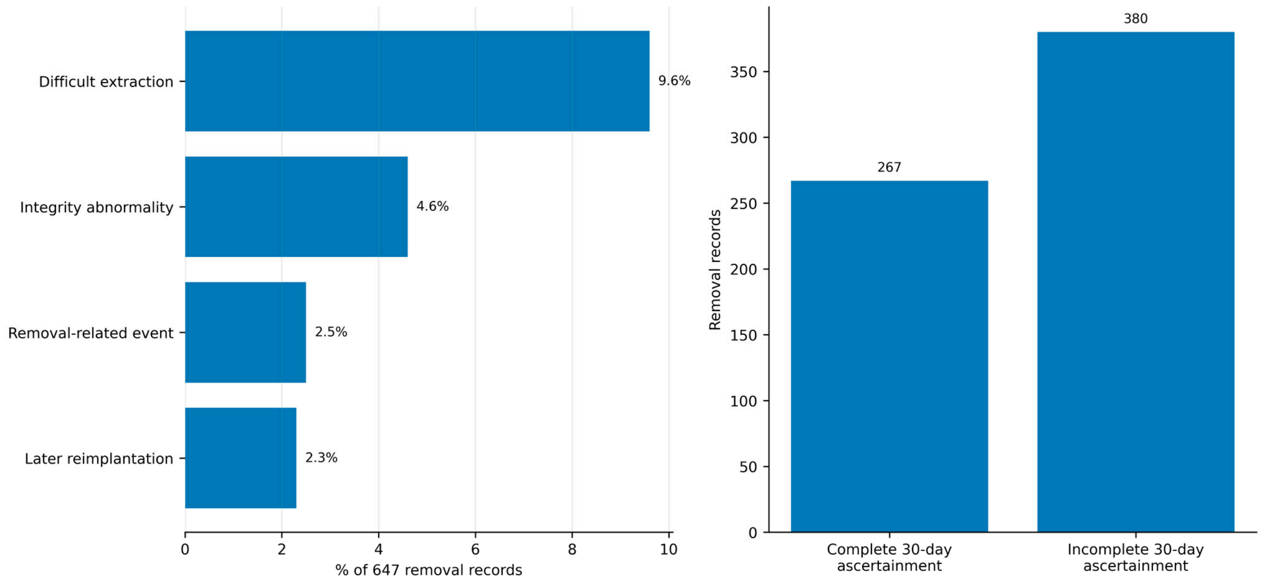

Supplementary Figure S3. Record-based removal outcomes and completeness of post-removal outcome ascertainment. (A) Documented procedural findings among record-based TIVAP removal procedures, including difficult extraction, catheter integrity abnormalities, removal-related events, and later reimplantation. Removal records represent procedures rather than mutually exclusive patient-level first events because some patients underwent more than one removal-related procedure after prior TIVAP reactivation. (B) Completeness of 30-day post-removal outcome ascertainment. Records without complete follow-up were retained as incomplete ascertainment and were not included in estimation of an overall 30-day post-removal complication incidence.

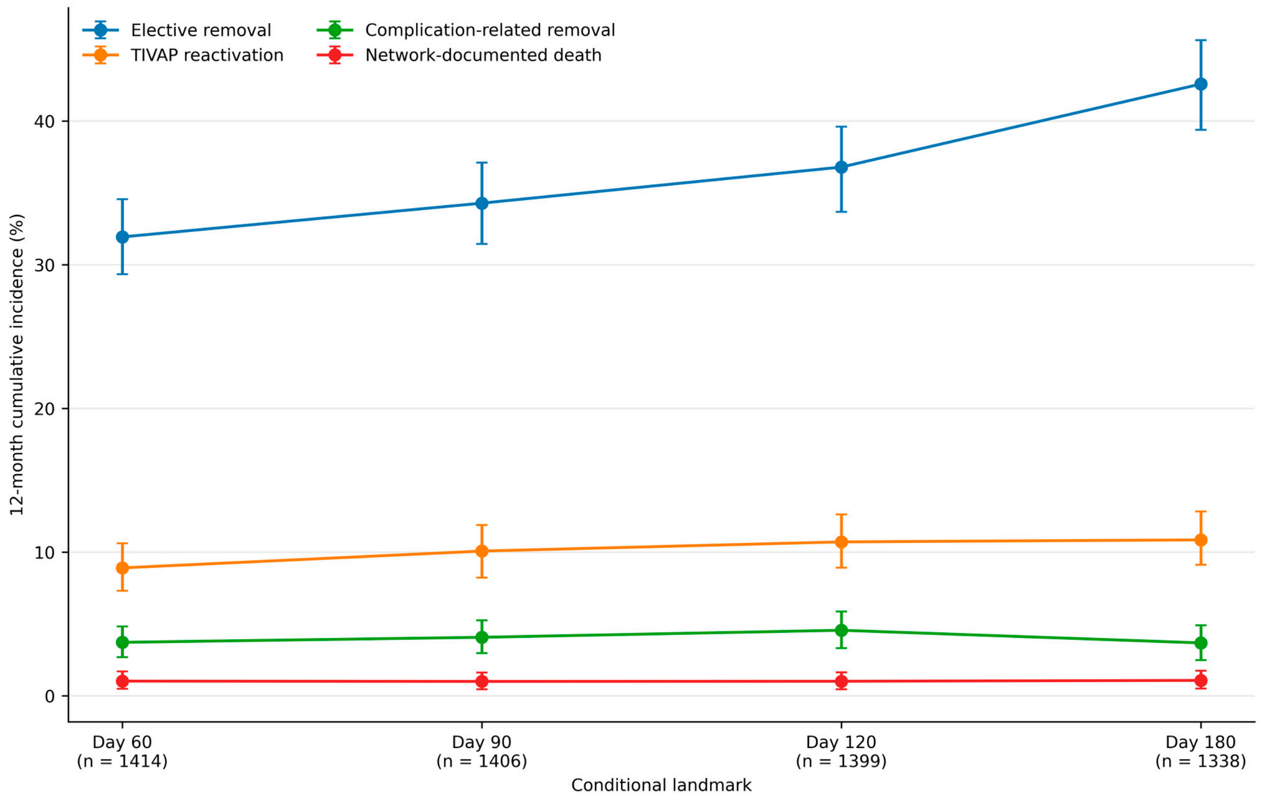

Supplementary Figure S4. Alternative conditional-landmark analyses of TIVAP management trajectories. The 12-month cumulative incidence of elective removal, TIVAP reactivation, complication-related removal, and network-documented death was independently reconstructed using alternative conditional landmarks at days 60, 90, 120, and 180 after the last qualifying intravenous anticancer treatment. Each landmark generated a separate risk set with eligibility reassessed at that time point; therefore, estimates represent different conditional cohorts rather than comparisons of reassessment strategies. Cumulative incidence estimates accounted for competing first events.

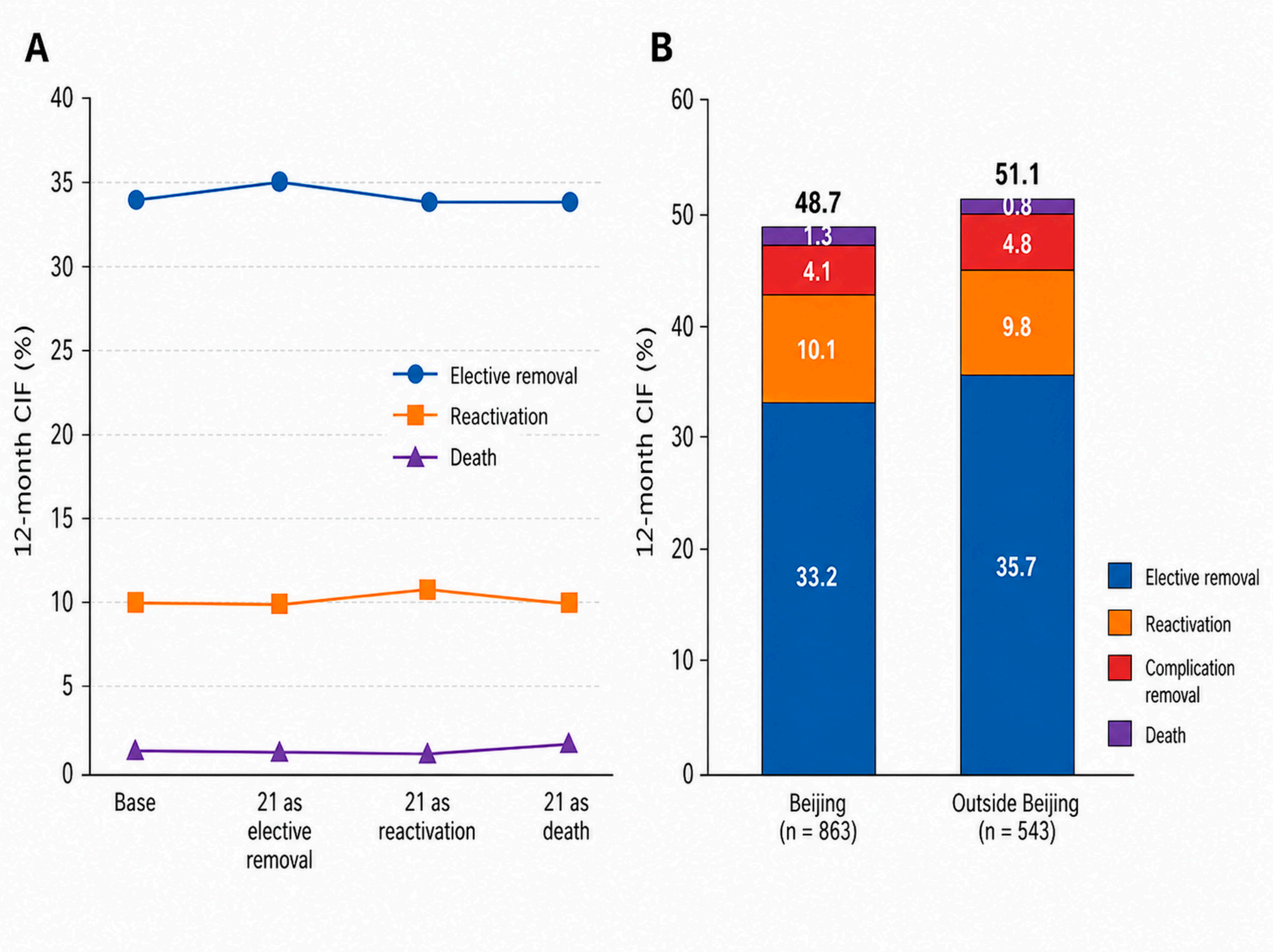

Supplementary Figure S5. Sensitivity analyses for unresolved external-event scenarios and residence-stratified cumulative incidence estimates. (A) Scenario analyses evaluated the potential influence of unresolved external events among censored records. Records without confirmed external-event status were assigned to elective removal, TIVAP reactivation, or death at the censoring date to generate bounded cumulative incidence estimates. These analyses represent sensitivity scenarios under extreme assumptions and do not indicate confirmed event occurrence. (B) Residence-stratified descriptive analyses compared 12-month cumulative incidence estimates among patients residing inside and outside Beijing. These analyses were descriptive and were not designed to establish geographic effects or causal associations.
